# Potential survival benefit of polymyxin B hemoperfusion in patients with septic shock: a propensity-matched cohort study

**DOI:** 10.1186/s13054-017-1712-3

**Published:** 2017-06-07

**Authors:** Yoshihiko Nakamura, Taisuke Kitamura, Fumiaki Kiyomi, Mineji Hayakawa, Kota Hoshino, Yasumasa Kawano, Reiko Yamasaki, Takeshi Nishida, Mariko Mizunuma, Hiroyasu Ishikura, Japan Septic Disseminated Intravascular Coagulation (JSEPTIC DIC) study group, Shinjiro Saito, Shigehiko Uchino, Daisuke Kudo, Toshihiko Mayumi, Takeo Azuhata, Fumihito Ito, Shodai Yoshihiro, Hayakawa Katsura, Tsuyoshi Nakashima, Takayuki Ogura, Eiichiro Noda, Yoshihiko Nakamura, Ryosuke Sekine, Kazuma Yamakawa, Yoshiaki Yoshikawa, Motohiro Sekino, Keiko Ueno, Yuko Okuda, Masayuki Watanabe, Akihito Tampo, Nobuyuki Saito, Yuya Kitai, Kohei Takimoto, Hiroki Takahashi, Iwao Kobayashi, Yutaka Kondo, Masamitsu Sanui, Yusuke Iizuka, Wataru Matsunaga, Sho Nachi, Toru Miike, Hiroshi Takahashi, Shuhei Takauji, Kensuke Umakoshi, Takafumi Todaka, Hiroshi Kodaira, Kohkichi Andoh, Takehiko Kasai, Yoshiaki Iwashita, Hideaki Arai, Masato Murata, Masahiro Yamane, Kazuhiro Shiga, Naoto Hori

**Affiliations:** 1 Department of Emergency and Critical Care Medicine, Faculty of Medicine, Fukuoka University, 7-45-1 Nanakuma, Jonan-ku, Fukuoka, 814-0180 Japan; 2 Academia, Industry and Government Collaborative Research Institute of Translational Medicine for Life Innovation, Fukuoka University, 7-45-1 Nanakuma, Jonan-ku, Fukuoka, 814-0180 Japan; 3 Emergency and Critical Care Center, Hokkaido University Hospital, N14W5, Kita-ku, Sapporo, 060-8648 Japan

**Keywords:** Polymyxin B hemoperfusion, Septic shock, Propensity score matching, Intensive care unit-free days, Mortality

## Abstract

**Background:**

The purpose of this study was to investigate whether polymyxin B hemoperfusion (PMX-HP) improves the survival of patients with septic shock.

**Methods:**

This was a retrospective, multicenter study conducted on patients treated during a 3-year period. We performed propensity-score analyses of the Japan Septic Disseminated Intravascular Coagulation (JSEPTIC DIC) study database. The study included data on 1723 patients with septic shock aged 16 years or older. Furthermore, we divided patients into to PMX-HP- and non-PMX-HP-treated groups. The primary endpoint was all-cause hospital mortality; secondary endpoints included intensive care unit (ICU) mortality and number of ICU-free days (ICUFDs) in the first 28 days.

**Results:**

Of 1,723 eligible patients, 522 had received PMX-HP. Propensity score matching created 262 matched pairs (i.e., 262 patients in each of the non-PMX-HP and PMX-HP groups). The proportion of all-cause hospital mortality was significantly lower in the PMX-HP group than in the non-PMX-HP group (32.8% vs. 41.2%; odds ratio (OR): 0.681; 95% confidence interval (CI): 0.470–0.987; *P* = 0.042). The number of ICUFD in the first 28 days was significantly higher in the PMX-HP group than in the non-PMX-HP group (18 (0-22) vs. 14 (0-22) days, respectively; *P* = 0.045). On the other hand, there was no significant difference in ICU mortality between the two groups (21.8% vs. 24.4%; OR: 0.844; CI: 0.548–1.300; *P* = 0.443).

**Conclusions:**

Our results strongly suggest that PMX-HP reduces all-cause hospital mortality and length of ICU stay in patients with septic shock.

**Electronic supplementary material:**

The online version of this article (doi:10.1186/s13054-017-1712-3) contains supplementary material, which is available to authorized users.

## Background

Despite the availability of modern antibiotics and resuscitation therapies, sepsis is a leading cause of death in critically ill patients [1]. Treatment of patients with septic shock is a major challenge for physicians. To improve clinical management and outcome of critically ill patients, the Surviving Sepsis Campaign guidelines were published approximately a decade ago and were most recently revised in 2012 [2].

Endotoxin, a lipopolysaccharide derived from the outer membranes of gram-negative rods (GNRs), is a key factor in the sepsis cascade because it triggers a series of inflammatory reactions that lead to organ dysfunction [3, 4]. Because high serum concentrations of endotoxin are closely linked to increased risk of multiple organ failure and death [5, 6], endotoxin is considered a therapeutic target in treating sepsis [7]. Polymyxin B direct hemoperfusion (PMX-HP) removes plasma endotoxins and is considered an effective treatment for sepsis [8]. Moreover, Totsugawa et al. [9] reported that PMX-HP not only removes plasma endotoxins, but also causes a drastic decrease in the doses of inotropic agents and a shortening of the duration of mechanical ventilation in patients with severe sepsis and/or septic shock from gram-positive cocci (GPC). Moreover, Yamato et al. [10] reported that treatment with a combination of PMX-HP and recombinant human soluble thrombomodulin (rhsTM) significantly improves survival rates after septic shock with disseminated intravascular coagulation (DIC) due to GPC or GNR infections. These results suggest that PMX-HP has a survival benefit not only in patients with GNR infections, but also in those with GPC-induced events. Two randomized controlled trials (RCTs) of abdominal septic shock have been reported to date. One is the EUPHAS trial [11], which reported a significant reduction in the 28-day mortality rate with PMX-HP in comparison with conventional treatment (32% vs. 53%; hazard ratio (HR): 0.36; 95% confidence interval (CI): 0.16–0.80; *P* = 0.01). In contrast, in the ABDOMIX RCT [12] there was no significant difference in the 28-day mortality rate between PMX-HP and conventional treatment (27.7% vs. 19.5%, respectively; *P* = 0.14). Therefore, it remains unclear whether PMX-HP produces a survival benefit in patients with abdominal septic shock. Furthermore, no studies have compared the usefulness of PMX-HP for various infection sites and different types of septic shock-causing pathogens as well as GNRs.

Therefore, we conducted this retrospective study in a large number of Japanese multi-intensive care unit (ICU) patients with septic shock arising from various sites of infection and types of pathogens to determine the efficacy of PMX-HP in reducing mortality using propensity score analysis.

## Methods

### Study design, setting, and selection of participants

This retrospective, observational study used the dataset of the Japan Septic Disseminated Intravascular Coagulation (JSEPTIC DIC) study, which was conducted in 42 ICUs in 40 institutions in Japan (Additional files 1 and 2) and was approved by the Institutional Review Boards of all participating hospitals. The JSEPTIC DIC study aimed to evaluate anti-DIC drugs in patients with severe sepsis or septic shock who were admitted to ICUs between January 2011 and December 2013 [13, 14]. Because this database had already been anonymized for individual patient data and institutions, the Institutional Review Board waived the need for review of this post-hoc study. However, we did not input patient personal data such as name or medical ID number at each facility in order to adhere strictly to the anonymity of patients. Included patients were those aged ≥16 years who had been admitted to the study ICUs between January 2011 and December 2013 for treatment of severe sepsis or septic shock, as defined by the International Sepsis Definitions Conference [15].

### Data collection

In this study, the following information was collected from the JSEPTIC DIC study dataset: age, sex, body weight (BW), type of ICU, route of admission to the ICU, ICU policy, number of ICU beds, pre-existing organ dysfunction, Acute Physiology and Chronic Health Evaluation (APACHE) II score [16], total Sequential Organ Failure Assessment (SOFA) score [17], systemic inflammatory response syndrome (SIRS) score [18], Japanese Association for Acute Medicine (JAAM) DIC score [19], primary infection site, microorganisms responsible for sepsis, laboratory tests (white blood cell count (WBC), platelet count, hemoglobin (Hb) and prothrombin time-international normalized ratio (PT-INR)) at the time of admission, packed red blood cells (PRBC) administered, surgical interventions at the infection site, anti-DIC drugs (rhsTM, antithrombin (AT) III products, protease inhibitors, or heparinoids), intravenous immunoglobulin (IVIG), low-dose steroids, and renal replacement therapy (RRT) for renal or non-renal indications, and PMX-HP during the first week after ICU admission. Furthermore, all-cause hospital mortality, ICU mortality, and length of ICU stay were collected for evaluation of the endpoints. We defined the ICU policy as follows: open ICU was defined as all patients admitted to the ICU were managed by each department of doctors; closed ICU was defined as all patients admitted to the ICU were managed by intensivists or anesthesiologists or emergency doctors.

### Patient selection

Patients with SOFA cardiovascular scores <3 and those who did not receive catecholamines upon ICU admission were excluded because they did not fit the criteria for septic shock. Furthermore, patients for whom the following data were missing were excluded: BW, SOFA score, WBC, Hb, platelet count, and PT-INR. Eligible patients were then allocated to PMX-HP and non-PMX-HP groups.

### PMX-HP

PMX-HP was performed with an adsorbent column designed for clinical use that contained 5 mg of PMX per gram of polystyrene fiber (Toray Industries, Tokyo, Japan) [20, 21]. This device was approved in 1994 and is widely used for treating severe sepsis in clinical settings in Japan [22].

### Endpoints

The primary endpoint of this study was all-cause hospital mortality, and the secondary endpoints ICU mortality and ICU-free days (ICUFDs). ICUFDs were calculated as follows: ICUFDs = 0 if the patient died during the first 28 days; ICUFDs = (28 − *x*) if the patient survived more than 28 days, where *x* is the number of days spent in the ICU; and ICUFDs = (28 − *y*) if the patient had been transferred to another hospital before 28 days had elapsed, where *y* is the number of days spent in the ICU.

### Statistical analysis

Data are expressed as number (%) for categorical variables, mean ± standard deviation (SD) for normally distributed variables, and median (first quartile to third quartile) for non-normally distributed variables. One-to-one nearest neighbor matching without replacement was performed between the PMX-HP and non-PMX-HP groups based on the estimated propensity scores for each patient. To estimate the propensity score, a logistic regression model was fitted for patients who had undergone PMX-HP treatment as a function of patient and ICU characteristics, including age, sex, BW, type of ICU, route of admission to the ICU, ICU policy, number of ICU beds, pre-existing organ dysfunction, APACHE II score, total SOFA, a SIRS score <2 vs. ≥2 [18], JAAM DIC score ≥4 vs. <4 [19], primary infection site, microorganisms responsible for sepsis, laboratory tests (WBC, platelet count, Hb concentration, and PT-INR), PRBC administration, surgical interventions at the infection site, anti-DIC drugs (rhsTM, AT III products, protease inhibitors, or heparinoids), IVIG, low-dose steroids, and RRT for renal or non-renal indications. A caliper width equal to 0.01 of the standard deviation of the logit of the propensity score was used. The standardized difference was used to evaluate covariate balance. An absolute standardized difference of >10% signifies a meaningful imbalance [23].

To evaluate differences between the PMX-HP and non-PMX-HP groups, categorical variables were compared by logistic regression, whereas continuous variables were compared by Student’s *t* tests or Wilcoxon test. ICU and hospital mortality rates were analyzed using conditional logistic regression, including the group (PMX-HP vs. non-PMX-HP) as a covariate and the matched set as a stratum. A signed rank test was used to compare the ICUFDs between groups. SAS version 9.4 (SAS Institute, Cary, NC, USA) was used for all analyses.

## Results

### Patients

**Fig. 1:**
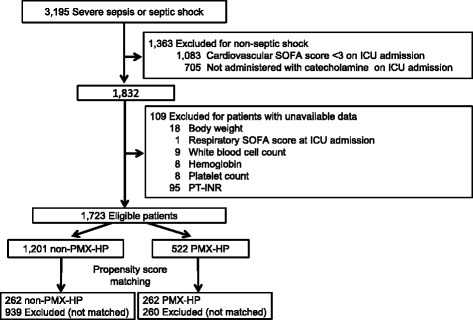
Patient selection schema. *ICU* intensive care unit, *PMX-HP* polymyxin B hemoperfusion, *PT-INR* prothrombin time-international normalized ratio, *SOFA* Sequential Organ Failure Assessment

This study enrolled 3,195 patients over the observational period, 1,363 of whom did not have septic shock and were excluded, as were 109 patients for whom the required data were unavailable. Finally, the 1,723 eligible patients were categorized into the PMX-HP (*n* = 522) or non-PMX-HP (control group; *n* = 1,201) groups, from which 262 propensity score-matched pairs were generated (Fig. 1). The *C* statistic indicated that the goodness of fit was 0.849 in the propensity score model.

The baseline characteristics of the unmatched PMX-HP and non-PMX-HP groups and those of the propensity score-matched groups are shown in Tables 1 and 2. When the unmatched groups were compared, ICU type (emergency center or surgical ICU), route of admission to the ICU, ICU policy, number of ICU beds, pre-existing organ dysfunction, SOFA score, JAAM DIC, primary infection site, microorganism, WBC, platelet counts, Hb, PT-INR, PRBC, surgical intervention, and other therapeutic interventions (rhsTM, AT III concentrate, protease inhibitors, IVIG, low-dose steroids, RRT, and non-renal RRT) differed significantly between the two groups. After propensity score matching, the baseline patient characteristics were well balanced between the groups, the standardized differences being ≤0.1.

**Table 1:** Relevant patient characteristics according to study group

Variables	Unmatched group					Matched group				
	All patients (*n* = 1,723)	non-PMX-HP (*n* = 1,201)	PMX-HP (*n* = 522)	SD	*P* value	All patients (*n* = 524)	non-PMX-HP (*n* = 262)	PMX-HP (*n* = 262)	SD	*P* value
Age, years	69.7 (13.8)	69.5 (14.0)	70.0 (13.1)	0.036	0.502	69.9 (13.1)	70.3 (13.4)	69.4 (12.8)	0.070	0.422
Male, *n* (%)	1019 (59.1)	705 (58.7)	314 (60.2)	0.030	0.574	304 (58.0)	150 (57.3)	154 (58.8)	0.031	0.723
Body weight, kg	56.6 (14.1)	56.6 (14.5)	56.6 (13.4)	0.007	0.903	56.5 (14.6)	56.0 (14.4)	57.0 (14.8)	0.064	0.465
Emergency center ICU, *n* (%)	729 (42.3)	486 (40.5)	243 (46.6)	0.123	0.019	239 (45.6)	121 (46.2)	118 (45.0)	0.023	0.793
Admission route to the ICU, *n* (%)										
Emergency department	735 (42.7)	543 (45.2)	192 (36.8)	0.172	0.000	215 (41.0)	109 (41.6)	106 (40.5)	0.023	0.790
Other hospital	423 (24.6)	287 (23.9)	136 (26.1)	0.050	0.339	121 (23.1)	62 (23.7)	59 (22.5)	0.027	0.756
Hospital ward	565 (32.8)	371 (30.9)	194 (37.2)	0.133	0.011	188 (35.9)	91 (34.7)	97 (37.0)	0.048	0.585
ICU policy, *n* (%)										
Open ICU	549 (31.9)	387 (32.2)	162 (31.0)	0.026	0.628	160 (30.5)	74 (28.2)	86 (32.8)	0.100	0.255
Closed ICU	897 (52.1)	637 (53.0)	260 (49.8)	0.065	0.217	281 (53.6)	147 (56.1)	134 (51.1)	0.100	0.255
Others	277 (16.1)	177 (14.7)	100 (19.2)	0.118	0.022	83 (15.8)	41 (15.6)	42 (16.0)	0.011	0.905
Number of ICU beds, number	12 (8–18)	12 (10–19)	10 (7–12)	0.429	<0.001	10.5 (8–16)	12 (8–15)	10 (7–16)	0.041	0.361
Pre-existing organ dysfunction, *n* (%)										
Liver insufficiency	83 (4.8)	49 (4.1)	34 (6.5)	0.109	0.032	30 (5.7)	12 (4.6)	18 (6.9)	0.099	0.262
Chronic respiratory disorder	66 (3.8)	46 (3.8)	20 (3.8)	0.000	1.000	18 (3.4)	6 (2.3)	12 (4.6)	0.126	0.158
Chronic heart failure	99 (5.7)	68 (5.7)	31 (5.9)	0.012	0.821	29 (5.5)	13 (5.0)	16 (6.1)	0.050	0.567
Chronic hemodialysis	149 (8.6)	99 (8.2)	50 (9.6)	0.047	0.365	53 (10.1)	25 (9.5)	28 (10.7)	0.038	0.664
Immunocompromised	299 (17.4)	218 (18.2)	81 (15.5)	0.070	0.185	83 (15.8)	43 (16.4)	40 (15.3)	0.031	0.720
None	1,108 (64.3)	773 (64.4)	335 (64.2)	0.004	0.941	338 (64.5)	177 (67.6)	161 (61.5)	0.128	0.145
Severity										
APACHE II score	25.4 (8.9)	25.2 (8.9)	25.9 (8.9)	0.083	0.115	25.2 (9.2)	25.5 (8.9)	25.0 (9.5)	0.060	0.493
SOFA score	11.5 (3.5)	11.2 (3.5)	12.0 (3.5)	0.246	<0.001	11.6 (3.4)	11.7 (3.4)	11.5 (3.4)	0.067	0.442
SIRS positive^a^, *n* (%)	1,654 (96.0)	1151 (95.8)	503 (96.4)	0.027	0.611	499 (95.2)	253 (96.6)	246 (93.9)	0.126	0.157
JAAM DIC positive^b^, *n* (%)	1,092 (63.4)	723 (60.2)	369 (70.7)	0.222	<0.001	345 (65.8)	176 (67.2)	169 (64.5)	0.056	0.519

Data are presented as mean (standard deviation), median (first quartile to third quartile), or number (percentage)

^a^SIRS criteria ≥2

^b^JAAM DIC score ≥4

*APACHE* Acute Physiology and Chronic Health Evaluation, *DIC* disseminated intravascular coagulation, *ICU* intensive care unit, *JAAM* Japanese Association for Acute Medicine, *PMX-HP* polymyxin B hemoperfusion, *SD* standardized difference, *SIRS* systemic inflammatory response syndrome, *SOFA* Sequential Organ Failure Assessment

**Table 2:** Characteristics of patients, laboratory findings, and treatment for sepsis according to study group

Variables	Unmatched group					Matched group				
	All patients (*n* = 1,723)	non-PMX-HP (*n* = 1,201)	PMX-HP (*n* = 522)	SD	*P* value	All patients (*n* = 524)	non-PMX-HP (*n* = 262)	PMX-HP (*n* = 262)	SD	*P* value
Primary infection site, *n* (%)										
Abdomen	618 (35.9)	321 (26.7)	297 (56.9)	0.642	<0.001	226 (43.1)	113 (43.1)	113 (43.1)	0.000	1.000
Lung/thorax	385 (22.3)	323 (26.9)	62 (11.9)	0.387	<0.001	93 (17.7)	47 (17.9)	46 (17.6)	0.010	0.909
Urinary tract	268 (15.6)	189 (15.7)	79 (15.1)	0.017	0.751	97 (18.5)	46 (17.6)	51 (19.5)	0.049	0.574
Bone/soft tissue	192 (11.1)	147 (12.2)	45 (8.6)	0.119	0.029	52 (9.9)	28 (10.7)	24 (9.2)	0.051	0.559
Cardiovascular	31 (1.8)	27 (2.2)	4 (0.8)	0.122	0.043	7 (1.3)	3 (1.1)	4 (1.5)	0.033	0.705
Central nervous system	26 (1.5)	25 (2.1)	1 (0.2)	0.179	0.019	1 (0.2)	0 (0.0)	1 (0.4)	0.088	–
Catheter-related	30 (1.7)	27 (2.2)	3 (0.6)	0.142	0.024	6 (1.1)	4 (1.5)	2 (0.8)	0.072	0.421
Others	34 (2.0)	27 (2.2)	7 (1.3)	0.068	0.219	8 (1.5)	3 (1.1)	5 (1.9)	0.062	0.481
Unknown	139 (8.1)	115 (9.6)	24 (4.6)	0.195	0.001	34 (6.5)	18 (6.9)	16 (6.1)	0.031	0.723
Microorganisms, *n* (%)										
Gram-negative bacteria	656 (38.1)	429 (35.7)	227 (43.5)	0.159	0.002	230 (43.9)	113 (43.1)	117 (44.7)	0.031	0.725
Gram-positive coccus	402 (23.3)	311 (25.9)	91 (17.4)	0.207	<0.001	105 (20.0)	50 (19.1)	55 (21.0)	0.048	0.585
Fungus	23 (1.3)	18 (1.5)	5 (1.0)	0.049	0.373	8 (1.5)	5 (1.9)	3 (1.1)	0.062	0.481
Mixed infection	232 (13.5)	142 (11.8)	90 (17.2)	0.154	0.003	72 (13.7)	39 (14.9)	33 (12.6)	0.067	0.447
Others	29 (1.7)	20 (1.7)	9 (1.7)	0.005	0.930	9 (1.7)	3 (1.1)	6 (2.3)	0.088	0.323
Unknown	371 (21.5)	272 (22.6)	99 (19.0)	0.091	0.088	100 (19.1)	52 (19.8)	48 (18.3)	0.039	0.657
Laboratory tests on admission to the ICU										
WBC, 10^9^/L	10.8 (3.6–17.7)	11.8 (5.0–18.1)	7.4 (2.2–16.1)	0.188	<0.001	10.4 (3.4–18.5)	11.6 (4.6–18.1)	9.7 (2.7–18.8)	0.012	0.195
Platelet counts, 10^9^/L	108 (58–174)	113 (60–186)	99 (53–154)	0.189	<0.001	100 (54–164)	92 (52–162)	108 (57–167)	0.100	0.126
Hb, g/L	10.5 (2.5)	10.6 (2.5)	10.4 (2.4)	0.099	0.063	10.4 (2.5)	10.3 (2.7)	10.5 (2.4)	0.081	0.355
PT-INR	1.4 (1.2–1.7)	1.4 (1.2–1.6)	1.5 (1.3–1.8)	0.122	<0.001	1.4 (1.3–1.8)	1.4 (1.2–1.8)	1.4 (1.3–1.8)	0.091	0.477
PRBC administration, *n* (%)	889 (51.6)	546 (45.5)	343 (65.7)	0.416	<0.001	313 (59.7)	159 (60.7)	154 (58.8)	0.039	0.656
Surgical intervention, *n* (%)	795 (46.1)	450 (37.5)	345 (66.1)	0.598	<0.001	289 (55.2)	146 (55.7)	143 (54.6)	0.023	0.792
Other therapeutic intervention, *n* (%)										
rhsTM	571 (33.1)	329 (27.4)	242 (46.4)	0.401	<0.001	203 (38.7)	101 (38.5)	102 (38.9)	0.008	0.929
AT III concentrate	653 (37.9)	353 (29.4)	300 (57.5)	0.591	<0.001	241 (46.0)	121 (46.2)	120 (45.8)	0.008	0.930
Protease inhibitors	209 (12.1)	117 (9.7)	92 (17.6)	0.231	<0.001	64 (12.2)	33 (12.6)	31 (11.8)	0.023	0.790
Heparinoids	69 (4.0)	44 (3.7)	25 (4.8)	0.056	0.275	23 (4.4)	11 (4.2)	12 (4.6)	0.019	0.831
IVIG	619 (35.9)	360 (30.0)	259 (49.6)	0.410	<0.001	224 (42.7)	117 (44.7)	107 (40.8)	0.077	0.377
Low-dose steroid	568 (33.0)	333 (27.7)	235 (45.0)	0.365	<0.001	196 (37.4)	101 (38.5)	95 (36.3)	0.047	0.588
RRT	627 (36.4)	352 (29.3)	275 (52.7)	0.489	<0.001	245 (46.8)	121 (46.2)	124 (47.3)	0.023	0.793
Non-renal indication RRT	189 (11.0)	45 (3.7)	144 (27.6)	0.694	<0.001	53 (10.1)	26 (9.9)	27 (10.3)	0.013	0.885

Data are presented as mean (standard deviation), median (first quartile to third quartile), or number (percentage)

*AT* antithrombin, *Hb* hemoglobin, *IVIG* intravenous immunoglobulins, *PMX-HP* polymyxin B hemoperfusion, *PRBC* packed red blood cells, *PT-INR* prothrombin time-international normalized ratio, *rhsTM* recombinant human soluble thrombomodulin, *RRT* renal replacement therapy, *SD* standardized difference, *WBC* white blood cell count

### Endpoints

The overall all-cause hospital mortality was 37.0% (637/1,723). There was no significant difference in all-cause hospital mortality between the two unmatched groups (PMX-HP vs. non-PMX-HP: 37.9% vs. 36.6%, respectively; odds ratio (OR): 1.061; 95% CI: 0.858–1.312; *P* = 0.585). However, a significant difference was observed between the two groups after propensity-score matching (PMX-HP vs. non-PMX-HP: 32.8% vs. 41.2%, respectively; OR: 0.681; 95% CI: 0.470–0.987; *P* = 0.042). Additionally, in the propensity-score matched groups, number of ICUFDs in the first 28 days was significantly greater in the PMX-HP group than in the non-PMX-HP group (18 (0–22) vs. 14 (0–22) days, respectively; *P* = 0.045). On the other hand, there was no significant difference in ICU mortality between the two groups (21.8% vs. 24.4%, respectively; OR: 0.844; 95% CI: 0.548–1.300; *P* = 0.443) (Table 3).

**Table 3:** Mortality and number of IUCFDs in the propensity-matched groups analyses

Variables	Unmatched group						Matched group					
	non-PMX-HP (*n* = 1201)	PMX-HP (*n* = 522)	OR	Difference	95% CI	*P* value	non-PMX-HP (*n* = 262)	PMX-HP (*n* = 262)	OR	Difference	95% CI	*P* value
All-cause hospital mortality, *n* (%)	439 (36.6)	198 (37.9)	1.061		(0.858–1.312)	0.585	108 (41.2)	86 (32.8)	0.681		(0.470–0.987)	0.042
ICU mortality, *n* (%)	268 (22.3)	128 (24.5)	1.131		(0.889–1.440)	0.317	64 (24.4)	57 (21.8)	0.844		(0.548–1.300)	0.443
28 ICUFDs (days)	16 (0–23)	15 (0–21)		0.0	(0.0–0.0)	0.255	14 (0–22)	18 (0–22)		1.5	(0.0–3.5)	0.045

Data are presented as median (first quartile to third quartile) or number (percentage)

In the unmatched group, categorical variables were compared by logistic regressions, and continuous variables were compared by Wilcoxon tests and Hodges-Lehmann estimates were presented for difference and 95% CI. In the matched group, all-cause hospital mortality and ICU mortality were analyzed by conditional logistic regression including PMX-DHP as a covariate and matched-set as a stratum, and ICUFDs were analyzed by signed rank test and Hodges-Lehmann estimate was presented for difference and 95% CI

*CI* confidence interval, *ICU* intensive care unit, *ICUFDs* ICU-free days, *OR* odds ratio, *PMX-HP* polymyxin B hemoperfusion

## Discussion

Our study included the largest number of patients with septic shock until now across 42 Japanese ICUs. PMX-HP has been accepted by the Japanese national health insurance program since 1994 [20]; more than 100,000 patients have received this treatment since then [24]. In Japan, PMX-HP is generally administered for severe sepsis or septic shock due to GNR infection (or suspected infection). In this study, we enrolled patients with septic shock associated with various sites of infection and pathogens; our data revealed that all-cause hospital mortality was significantly lower and there were significantly more ICUFDs in the PMX-HP group than in the non-PMX-HP group. Additionally, this is the first study to show a survival benefit of PMX-HP in patients with septic shock at various sites of infection and pathogens; these data are thus very noteworthy. Because various baseline characteristics differed between the PMX-HP (*n* = 522) and non-PMX-HP (*n* = 1201) groups, comparing all data for these two regimens would not be valid. Therefore, to adjust for differing baseline characteristics, we extracted 262 comparable subjects from each group by propensity score matching. Almost all baseline characteristics were homogenous between the resultant groups, making a comparison between them valid. Thus, the most valid conclusions can be derived from comparing the PMX-HP (*n* = 262) and non-PMX-HP (*n* = 262) groups.

Cruz et al. [11] have reported that PMX-HP produces improvements in mortality, as well as in cardiac index, mean arterial pressure, inotropic score, vasopressor dependency index, and mean PaO_2_/FiO_2_ ratio. Moreover, a recent large retrospective study [25] showed that PMX-HP treatment reduces 28-day mortality in high-risk patients with septic shock complicated by continuous RRT-requiring acute kidney injury (the 28-day mortality was 40.2% (393/978) in the PMX-HP group and 46.8% (458/978) in the non-PMX-HP group; *P* = 0.003). In contrast, using propensity-matched analysis of data from Japanese Diagnosis Procedure Combination (DPC) databases, Iwagami et al. [22] reported no significant survival benefit in patients with postoperative abdominal septic shock (the 28-day mortality was 17.1% (101/590) in the PMX-HP group and 16.3% (96/590) in the non-PMX-HP group; *P* = 0.696). However, their DPC database [22] did not incorporate the scoring systems generally used in critically ill patients (such as the APACHE II, SOFA, JAAM DIC, and SIRS scores). Therefore, we could not determine the severity of the patients’ conditions in their study. Additionally, they accepted patients who had received noradrenaline and/or dopamine on day 0 as possibly having septic shock. However, Hashiguchi and Iba [26] highlighted a lower 28-day mortality rate in their study than in previous studies [11, 27]. Because the JSEPTIC DIC study database does not supply 28-day mortality, we were unable to evaluate this variable. However, in our study, the mean APACHE II score was over 25 on entry and the overall all-cause hospital mortality rate was approximately 37% (194/524) in the matched group. Therefore, we strongly suspect that this discrepancy in mortality rates is attributable to differences in severity of illness between our study subjects and previous studies; that is, patients enrolled in the Iwagami et al. [22] study were possibly less severely ill than our patients. Furthermore, the hospital mortality rate was very high in both groups in this study. However, a previous Japanese cohort study reported a similar hospital mortality from septic shock (41.5%, 117/282) [28].

Regarding the curative effects of PMX-HP, a meta-analysis by Cruz et al. [29] reported that PMX-HP treatment is associated with an increase in mean arterial pressure of 19 mm Hg (95% CI: 15–22 mmHg; *P* < 0.001) and a decrease in dopamine/dobutamine dose of 1.8 μg/kg per minute (95% CI: 0.4–3.3 μg/kg per minute; *P* = 0.01). Furthermore, the mean PaO_2_/FiO_2_ ratio reportedly increases by 32 units (95% CI: 23–41 units; *P* < 0.001). These data suggest that PMX-HP improves patient hemodynamics and oxygenation. In our study, the PMX-HP group had significantly more ICUFDs in the first 28 days than the non-PMX-HP group; this result may be attributable to clinical effects similar to those reported by Cruz et al. [29].

In this study, organisms were isolated from a relatively high proportion of patients and subjected to microbiologic testing. In the previous Japanese Sepsis Registry database [30] a high percentage of patients had blood cultures (81.4%). Additionally, in the JSEPTIC DIC study, blood cultures were performed in 94% of patients [14]. Thus, microbiological tests can usually be performed in patients with sepsis in Japanese ICUs in accordance with the Japanese guidelines for the management of sepsis [31]. Moreover, we enrolled patients with various sites of infection and types of pathogen. Patients with GPC infection comprised 20.0% (205/524) of the matched group. Nevertheless, all-cause hospital mortality was significantly better in the PMX-HP group than in the non-PMX-HP group. PMX-HP was originally developed for removal of endotoxin and used to treat GNR-induced septic shock. However, our results suggest that PMX-HP also has a beneficial effect on GPC-induced septic shock. PMX-HP is reportedly able to adsorb endogenous cannabinoids such as anandamide and 2-arachidonoylglycerol [32], as well as activated monocytes [33]. The interaction of cannabinoids with vascular cannabinoid receptors leads to the hypotension that occurs in hemorrhagic or endotoxic shock [34–36]. Moreover, sepsis-induced immunoparalysis appears to play a key role in sepsis-induced morbidity and mortality [37]. One of the most important biomarkers of immunoparalysis is the human leukocyte antigen (HLA)-DR on the cell surface of monocytes (mHLA-DR), which is correlated with mortality [38, 39]. Ono et al. [40] reported that mHLA-DR was markedly decreased in patients with septic shock, and that this decrease was significantly reversed after PMX-HP treatment (*P* < 0.01). They thus concluded that PMX-HP may be a new strategy for helping patients to recover from immunoparalysis in septic conditions. In addition to endotoxin adsorption, adsorption of mediators such as endogenous cannabinoids or recovery from immunoparalysis may have contributed to the improvement in hospital mortality identified in this present study. In contrast, the JSEPTIC DIC study database does not contain information on cause of death and there is no clear explanation for the reported discrepancy between ICU mortality and all-cause hospital mortality. Hotchkiss et al. [41] reported development of immunoparalysis in later phases of sepsis. Hence, even though PMX-HP is an acute intervention, the hospital mortality (longer term mortality) observed in the present study may represent a significant improvement.

Some limitations of our study deserve consideration. First, this study was retrospective. Second, we did not consider the number of times (once or twice), the duration, or the initiation time of PMX-HP administration after ICU admission. Third, we did not examine long-term prognosis (such as 60-day or 90-day mortality). Fourth, a new definition of sepsis was published in 2016 [42]. Because the JSEPTIC DIC study was planned in November 2015, we used the 2001 consensus sepsis definition [15] in this study. Finally, we were unable to determine the exact timing of the various therapeutic interventions. However, because other therapeutic interventions and PMX-HP are usually performed simultaneously upon admission to the ICU, we considered it acceptable to use therapeutic interventions when estimating propensity scores. Because of these limitations, further studies (particularly RCTs) are required to more reliably ascertain the survival benefit of PMX-HP. In fact, the efficacy of PMX-HP treatment for septic shock is currently being investigated in the USA and Canada [43]; the results of these trials are eagerly anticipated.

## Conclusion

This study demonstrated that PMX-HP is associated with reduced all-cause hospital mortality and number of ICUFDs in patients with septic shock caused by various pathogens and with various sites of infection.

## Key messages

- Despite the availability of modern antibiotics and resuscitation therapies, sepsis is a leading cause of death in critically ill patients.
- Endotoxin, a lipopolysaccharide derived from the outer membranes of gram-negative rods, is a key factor in the sepsis cascade and high serum concentrations of endotoxin are closely linked to increased risk of multiple organ failure and death.
- Polymyxin B direct hemoperfusion (PMX-HP) removes plasma endotoxins and is considered an effective treatment for sepsis.
- The aim of this study was to investigate the usefulness of PMX-HP for various infection sites and different types of septic shock caused by not only gram-negative rods but other pathogens.
- This study included the largest number of patients with septic shock until now across 42 Japanese ICUs.
- PMX-HP is associated with reduced all-cause hospital mortality and number of ICU free days in patients with septic shock caused by various pathogens and with various sites of infection.

## Additional files

Additional file 1: Table S1. List of participating institutions (DOC 39 kb).

Additional file 2: Table S2. Characteristics of the intensive care units (ICUs). Data are presented as number (percentage) (DOC 29 kb).

## Abbreviations

APACHE: Acute Physiology and Chronic Health Evaluation
AT: Antithrombin
BW: Body weight
CI: Confidence interval
DIC: Disseminated intravascular coagulation
DPC: Diagnosis procedure combination
GNR: Gram-negative rod
GPC: Gram-positive cocci
Hb: Hemoglobin
HLA: Human leukocyte antigen
HR: Hazard ratio
ICU: Intensive care unit
ICUFDs: ICU-free days
IVIG: Intravenous immunoglobulin
JAAM: Japanese Association for Acute Medicine
JSEPTIC DIC: The Japan Septic Disseminated Intravascular Coagulation
OR: Odds ratio
PMX-HP: Polymyxin B hemoperfusion
PRBC: Packed red blood cells
PT-INR: Prothrombin time-international normalized ratio
RCT: Randomized controlled trial
rhsTM: Recombinant human soluble thrombomodulin
RRT: Renal replacement therapy
SIRS: Systemic inflammatory response syndrome
SOFA: Sequential Organ Failure Assessment
WBC: White blood cell count
