# Supplementary material for: Potential survival benefit of polymyxin B hemoperfusion in patients with septic shock: a propensity-matched cohort study
Source: Crit Care. 2017 Jun 7;21:134. doi: 10.1186/s13054-017-1712-3 (PMC5463489; doi:10.1186/s13054-017-1712-3)
Supplement: Supplementary file 1 — List of participating institutions (DOC 39 kb). [file 13054_2017_1712_MOESM1_ESM.doc]

**Additional file 1**

Supplemental Table 1. List of participating institutions

| Akashi City Hospital |
| --- |
| Asahikawa Medical University |
| Asahikawa Red Cross Hospital |
| Ehime University Hospital |
| Fukuoka University Hospital |
| Gifu University Hospital |
| Graduate School of Medicine, University of the Ryukyus |
| Gunma university |
| Hakodate Municipal Hospital |
| Hokkaido University Hospital |
| Hyogo College of Medicine |
| Ibaraki Prefectural Central Hospital |
| JA Hiroshima General Hospital |
| Japan Red Cross Maebashi Hospital |
| Jichi Medical University Saitama Medical Center |
| Jikei university school of medicine |
| Kameda Medical Center |
| KKR Sapporo Medical Center |
| Kyoto First Red-Cross Hospital |
| Kyushu University Hospital |
| Mie University Hospital |
| Nagasaki University Hospital |
| Nihon University School of Medicine |
| Nippon Medical School Chiba Hokusoh Hospital |
| Ohta General Hospital Foundation Ohta Nishinouchi Hospital |
| Osaka General Medical Center |
| Osaka University Hospital |
| Saga University Hospital |
| Saiseikai Yokohamasi Tobu Hospital |
| Saitama Red Cross Hospital |
| Sapporo City General Hospital |
| Seirei Mikatahara General Hospital |
| Sendai City Hospital |
| Shonan Kamakura General Hospital |
| Steel Memorial Muroran Hospital |
| Tohoku University Hospital |
| Tokyo Medical University, Hachioji Medical Center |
| Tomishiro Central Hospital |
| University of Occupational and Environmental Health Hospital |
| Wakayama Medical University Hospital |
