# Supplementary material for: Potential survival benefit of polymyxin B hemoperfusion in patients with septic shock: a propensity-matched cohort study
Source: Crit Care. 2017 Jun 7;21:134. doi: 10.1186/s13054-017-1712-3 (PMC5463489; doi:10.1186/s13054-017-1712-3)
Supplement: Supplementary file 2 — Characteristics of the intensive care units (ICUs). Data are presented as number (percentage) (DOC 29 kb). [file 13054_2017_1712_MOESM2_ESM.doc]

**Additional file** 2

Supplemental Table 2. Characteristics of the intensive care units

| ICU type |  |
| --- | --- |
| General ICU | 24 (57.1) |
| Emergency center ICU | 18 (42.9) |
| ICU policy |  |
| Closed policy | 17 (40.5) |
| Open policy | 18 (42.9) |
| Other | 7 (16.7) |

ICU, intensive care unit

Data are presented as number (percentage)
